# A survey of educator perspectives toward teaching harm reduction cannabis education

**DOI:** 10.1371/journal.pone.0299085

**Published:** 2024-05-08

**Authors:** Molly K. Downey, Lisa D. Bishop, Jennifer R. Donnan, Emily C. Rowe, Nick Harris

**Affiliations:** 1 Department of Psychology, Memorial University of Newfoundland, St. John’s, NL, Canada; 2 School of Pharmacy, Memorial University of Newfoundland, St. John’s, NL, Canada; 3 Faculty of Medicine, Memorial University of Newfoundland, St. John’s, NL, Canada; Chiba Daigaku, JAPAN

## Abstract

**Introduction:**

Substance use is common among youth which can adversely affect youth health. Despite the legalization of cannabis in Canada and much of the United States, there is a lack of harm reduction cannabis education in schools. In addition, educators may not feel prepared to teach students about cannabis.

**Methods:**

A cross-sectional survey explored educator perceptions toward teaching harm reduction substance use education to students in grades 4–12. Data analysis included descriptive statistics to evaluate demographic variables, ANOVAs to identify subgroup differences, and inductive thematic analysis to establish themes from open-ended responses. From the sample of 170 educators, the majority were female (77%) and worked as classroom teachers (59%).

**Results:**

Ninety-two percent of educators felt harm reduction was an effective approach to substance use education, and 84% stated that they would feel comfortable teaching cannabis harm reduction education to students. While 68% of educators believed they would be able to recognize if a student was under the influence of cannabis, only 39% felt certain about how to respond to student cannabis use, and just 8% felt that their current teacher training allowed them to intervene and prevent cannabis-related harms. Most educators (89%) expressed interest in harm reduction training, particularly interactive training (70%) and instructor-led lessons (51%). Online curriculum resources were preferred by 57%. Responses differed by gender and age group, with females of any age and educators under 40 reporting greater support of harm reduction approaches and more interest in training.

**Conclusion:**

Educators expressed considerable support for harm reduction substance use education, but many felt unprepared to address this topic with students. The findings identified a need for educator training on harm reduction substance use education, so that educators can help students make informed choices around substance use, thereby promoting youth health and safety.

## Introduction

Youth substance use is a significant public health concern in Canada and the United States and is associated with an increased risk of mental health issues [1] and substance use disorders [2]. Cannabis is the second most frequently consumed substance among youth in Canada and the United States, second only to alcohol [3, 4]. Non-medical cannabis use was legalized in Canada in 2018 and in 23 states in the United States as of June 2023 [5]. While a primary aim of cannabis legalization was to decrease youth access [6], cannabis use remains prevalent among Canadian adolescents. In 2022, 18.3% of Canadian students in grades 7–12 reported using cannabis in the past 12 months, with an average age of initiation of 14.1 years [3]. These findings are concerning given the diverse health risks associated with youth cannabis use, including increased rates of cognitive impairment [7], dependence [8], mental illness [9], respiratory issues [10] and motor vehicle collision [11].

Prior studies identified gaps in youth knowledge about the potential health and safety concerns of cannabis, including the impact on brain development and mental health, and the dangers of driving under the influence [12, 13]. Enhanced cannabis education can help close this knowledge gap and equip youth to make safe and informed choices, thereby protecting their health and safety. Research suggests that risk-based substance use education that calls for abstinence is ineffective, as this approach is inconsistent with many youths’ personal experiences [13, 14]. Many schools rely on external abstinence-focused programs such as the Drug Abuse Resistance Education (D.A.R.E.), which is largely unsuccessful at reducing substance use [15, 16] and has been described as ineffective by youth [12]. A harm reduction approach to substance use education is more realistic as it provides strategies to minimize related harms without requiring abstinence as the end goal [17–19].

Several studies examining the efficacy of harm reduction substance use education have yielded promising results. Poulin and Nicholson [20] examined the impact of a Canadian school-based harm reduction substance use education program, reporting that students in grades 10–12 who completed the program experienced significant decreases in heavy episodic drinking and driving while under the influence of cannabis. In a randomized controlled trial of a harm reduction cannabis education program in Australian high schools, Lester et al. [21] found that students in the experimental group reported significantly less cannabis-related harms and significantly smaller increases in cannabis consumption rates at three years post-test. Fischer [22] conducted a pilot study of an American school-based harm reduction program, reporting that participating high school students exhibited a significant increase in harm reduction knowledge and behaviours from pre-test to post-test.

In recent years, several resources have been published that encourage safer cannabis use practices using harm reduction strategies. The Lower-Risk Cannabis Use Guidelines [23] recommends strategies including avoiding or delaying use, choosing lower potency cannabis, purchasing legal products, avoiding driving after consumption, and avoiding poly-substance use. The Sensible Cannabis Education Toolkit [18] provides evidence-based guidance to parents and educators. Community programs have also emerged, including the Youth Cannabis Awareness Program [24], which offers free cannabis education to Canadian youth and supports school cannabis education initiatives.

Despite the availability of such resources, there are gaps in existing harm reduction cannabis education in Canadian schools [17, 25, 26]. Canadian provinces and territories are individually responsible for developing and providing health education, so there is no standardized cannabis education across the country [27]. In a post-cannabis legalization qualitative study exploring youths’ cannabis health literacy, youth expressed dissatisfaction with the scare tactic messaging employed by abstinence-based education and reported learning little about cannabis in school, often obtaining information from social media or peers [17]. In a recent scoping review and environmental scan that identified Canadian cannabis education resources [25], many resources were identified but there were issues with accessibility, quality and multicultural considerations. Most education resources were not formally evaluated, and there was limited school-wide programming. Although many resources were open access, educators may not use the materials as they have to independently identify them, assess their suitability, and implement them in the classroom. Research suggests that implementation may be further limited by educator time constraints and competing curriculum goals [28].

Educator attitudes and preparedness can also serve as barriers to implementing harm reduction education in the school system. The successful delivery of a health curriculum depends on the ongoing engagement of the educator, which can be influenced by personal attitudes, experience and values. Some educators have expressed the belief that cannabis education is best left to the student’s parents or healthcare providers, with over half stating that they had never been involved in cannabis education initiatives in the school [29]. Educators have reported feeling unprepared to discuss cannabis with students, partly due to gaps in their own knowledge. In one study of student cannabis use at a Canadian high school [28], educators identified being unfamiliar with the signs of cannabis use and how to identify and appropriately respond to student cannabis use. Educators expressed a need for teachers and school staff to receive proper cannabis education so that they could know the warning signs and how to best respond to student use.

### The current study

Our Cannabis Health Evaluation and Research Partnership team in Newfoundland and Labrador (NL), Canada is an interdisciplinary team that aims to understand the impacts of cannabis legalization and how individuals can be supported in making safe and informed choices [30]. We identified a goal of developing a harm reduction substance use education strategy for youth, including evidence-informed cannabis curriculum for students in grades 4–12. Exploring educator perspectives when developing a substance use education strategy is essential to preparing them to have informed, non-judgmental conversations with youth. Our study examined educator perspectives and needs around teaching harm reduction substance use education, particularly cannabis education. The insight gained from this research will help inform the development of a substance use education strategy that aligns with educator needs and preferences.

## Methods

Our study utilized a mixed-method approach. Research objectives included exploring 1) educator attitudes toward harm reduction education approaches, 2) educator needs regarding teaching cannabis education using a harm reduction approach and 3) educator preferences for receiving educator training and curriculum materials.

### Participants

Educators who worked in NL and had worked directly with students in grades 4–12 at any point since the 2018–2019 school year were eligible. The year 2018 was selected, as this was the first year since cannabis legalization in Canada. Educators included classroom teachers, principals, guidance counsellors, teacher-librarians, and teaching and learning assistants.

Recruitment was facilitated through targeted educator social media groups and email correspondence with school administrators, principals and teachers. The English and French school boards were contacted by email to request permission to conduct research in the province’s public schools, while private schools were recruited via direct correspondence with principals. We employed maximum variation sampling and invited all members of the target population to participate. Recruitment began on September 29, 2022, and continued until January 23, 2023.

A total of 205 educators consented to complete the survey. Thirty-four respondents were ineligible because they had not worked directly with students since 2018–2019 (*n* = 29) or were retired (*n* = 5). One additional respondent was excluded as they stopped answering survey questions after the demographic section. The final sample consisted of 170 educators, of which over three quarters identified as women (76.5%), and age ranged from under 25 to over 60. Most respondents were classroom teachers (58.8%) and held a permanent teaching position (69.4%). See Table 1 for more detailed information about sample demographics.

**Table 1:** Demographic characteristics of participants.

Demographic variable	*N* (%)
**Grades involved with since 2018** *	
10–12	108 (63.5)
7–9	117 (68.8)
4–6	85 (50.0)
**Primary teaching position**	
Classroom teacher	100 (58.8)
Instructional resource teacher	21 (12.4)
Speciality teacher (e.g., music, physical education)	14 (8.2)
Other	12 (7.1)
Principal/vice principal	8 (4.7)
Guidance counselor	7 (4.1)
Teacher-librarian	3 (1.8)
Teaching and learning assistant	3 (1.8)
Student assistant	2 (1.2)
**Employment status**	
Permanent	118 (69.4)
Replacement	26 (15.3)
Substitute	19 (11.2)
On-leave	7 (4.1)
**Age group**	
60+	4 (2.4)
50–59	34 (20.0)
40–49	43 (25.3)
30–39	57 (33.5)
25–29	21 (12.4)
Under 25	11 (6.5)
**Gender**	
Woman	130 (76.5)
Man	35 (20.6)
Gender Diverse	3 (1.8)
Decline to answer	2 (1.2)
**Years of teaching experience**	
21+	38 (22.4)
16–20	20 (11.8)
11–15	39 (22.9)
6–10	40 (23.5)
0–5	33 (19.4)
**Employer**	
English school board	161 (95.3)
French school board	5 (3.0)
Private school	3 (1.8)
**Primary teaching language**	
English	131 (77.1)
Both French and English	23 (13.5)
French	16 (9.4)
**Community size**	
Over 15,000	76 (45.0)
5000–15,000	38 (22.5)
Under 5000	55 (32.5)
**Number of students in school**	
Over 600	34 (20.1)
451–600	37 (21.9)
301–450	38 (22.5)
151–300	28 (16.6)
51–150	21 (12.4)
Under 50	11 (6.5)

Survey included 170 participants. Numbers may vary by question due to missing data.

*Respondents were asked to select all that apply.

### Measure

An online survey (S1 Appendix) was developed for this study to explore educator perspectives regarding their harm reduction substance use knowledge and perspectives. Survey items were constructed by drawing on a number of existing materials including a questionnaire assessing attitudes toward harm reduction [31], a survey examining teacher perspectives toward cannabis education [29], and several harm reduction resources [18, 23]. The survey was developed through iterative review in collaboration with experienced educators and school administrators, who verified that the final items were consistent with the target population’s knowledge, skills and experiences.

The survey contained four sections: 1) demographic information, 2) attitudes toward harm reduction and abstinence approaches to substance use education [29, 31], 3) perceived needs for the successful delivery of harm reduction cannabis education [23, 29], and 4) preferences for receiving cannabis education training and curriculum materials. The question format included multiple choice, rating scale, and open-ended items.

The terms *harm reduction* and *abstinence* were defined to ensure proper understanding. *Abstinence* was defined as the idea that avoiding drug use is the only acceptable option for everyone, while *harm reduction* was defined as an approach that recognizes that substance use among some youth is inevitable and focuses on decreasing the harms associated with substance use [18]. Participants were then provided with statements regarding harm reduction and abstinence and instructed to select the response that best reflected their personal opinion; response options were “strongly disagree,” “disagree,” “agree,” or “strongly agree.”

Educator needs in order to deliver harm reduction cannabis education were then assessed. Participant readiness to address cannabis use with students was indicated on a rating scale. Several multiple choice questions were adapted from the Lower-Risk Cannabis Use Guidelines [23] to test cannabis knowledge. For the cannabis knowledge questions, “I don’t know” was added as a response option to capture educators who were not confident in their knowledge. Other items included multiple choice questions about preferred means of receiving professional development, lesson plans and supplementary resources. Educators were also asked about their experiences with Mothers Against Drunk Driving (M.A.D.D.) and D.A.R.E. in their school by providing three words or phrases that described their perceptions of each of these programs [32, 33]. Finally, participants were asked two open-ended questions: “What other aspects of cannabis use should be addressed in the curriculum?” and “Please add any final thoughts that you would like to share regarding cannabis education in the school system.”

The survey included an option to be included in a prize draw, which was entered using a separate link to maintain the anonymity of survey responses. The prize draw option was omitted for those schools where the employer did not endorse prizes. The survey was conducted online using Qualtrics, where written informed consent was obtained. The survey took approximately 15 minutes to complete. The current study was approved by Memorial University’s Interdisciplinary Committee on Ethics in Human Research (ICEHR # 20230513-SC).

### Analysis

Descriptive statistics were used to examine the demographic characteristics of the sample and the frequency of responses to each survey item. Response frequencies varied based on missing data, with the number of missing responses per question ranging from zero to six. For cannabis knowledge-testing questions, responses for each question were coded as “correct,” “incorrect,” or “I don’t know” so that the frequency of correct responses could be calculated. Participant responses were categorized by age, gender, years of experience and community size. For the variable of gender, respondents who selected “gender diverse” (*n* = 3) or “other” (*n* = 2) were excluded from further group comparison analyses due to small sample sizes. The variable of age group was collapsed into two groups for data analysis, “39 and under” (*n* = 89) and “40 and over” (*n* = 81), as the over-60 group was extremely small (*n* = 4) and exhibited no variance on certain survey items. Independent-sample t-tests were conducted for gender and age group, and one-way ANOVAs were conducted for years of teaching experience and community size for each of the survey items. Games-Howell post-hoc tests were conducted for significant ANOVAs. Statistical analyses were performed using IBM SPSS Statistics (Version 28). A significance level of *α* = .05 was used when comparing the *p*-values for each test.

When analyzing the three words used to describe D.A.R.E. and M.A.D.D., words that were similar in meaning were combined into a single term. For example, the phrases “needs updating” and “in need of a revamp” were both coded as “outdated.” The frequency of each code was then determined to identify the most common descriptors for both programs.

An inductive thematic analysis was conducted for open-ended responses to identify potential themes. Data analysis was conducted manually using a line-by-line approach; initial codes were then analyzed to establish patterns in meaning, which were formed into themes. Three of the authors independently analyzed the data and then cross-referenced coding and emerging themes to ensure reliability across coders. Final themes were determined through an iterative review process completed by the three coders.

## Results

### Attitudes toward harm reduction substance use education

#### Personal attitudes toward harm reduction

Harm reduction was widely supported, with 92.2% of educators indicating that it was a practical and realistic approach to substance use education. Regarding the role of the school in harm reduction, 95.9% thought that students who use substances should have access to harm reduction supports within the school system. Almost all (95.9%) educators believed that access to evidence-based information on substances helps youth make safer choices. Interestingly, while 100% of educators felt that students should be given honest information about substance use-related harms, 15.8% believed that teaching youth about safer substance use would encourage them to use substances. Similarly, although 85.2% thought it is possible to use substances without misusing them, 46.3% indicated that students who use substances should be expected to pursue abstinence (Table 2).

**Table 2:** Personal attitudes toward harm reduction.

	Response N (%)			
Survey item	Strongly agree	Agree	Disagree	Strongly disagree
It is possible to use substances without misusing or abusing substances.	40 (23.7)	104 (61.5)	23 (13.6)	2 (1.2)
Students who use substances should be expected to pursue abstinence.	12 (7.3)	64 (39.0)	75 (45.7)	13 (7.9)
Students should be given honest information about how to avoid the harms associated with substance use.	141 (83.4)	28 (16.6)	0	0
Minimizing the risk of harm associated with substances should be discussed with students who seek help for substance use.	124 (72.9)	43 (25.3)	2 (1.2)	1 (0.6)
Students who use substances should have access to support services within the school system to help prevent harm.	122 (71.8)	41 (24.1)	3 (1.8)	4 (2.4)
Harm reduction is a practical, realistic approach that does not encourage substance use.	79 (47.6)	74 (44.6)	11 (6.6)	2 (1.2)
The “just say no” message regarding substance use is effective for many youths.	7 (4.1)	35 (20.7)	85 (50.3)	42 (24.9)
Access to evidence-based information on substances allows youth to make safer choices.	83 (48.8)	80 (47.1)	7 (4.1)	0
Abstinence-based education reduces harm associated with substance use among youth.	8 (4.8)	55 (33.3)	74 (44.8)	28 (17)
A harm reduction approach to substance use education can present abstinence to youth as an option without framing it as the only choice.	86 (51.5)	74 (44.3)	6 (3.6)	1 (0.6)
Teaching youth about safer substance use will encourage them to use substances.	5 (2.9)	22 (12.9)	104 (61.2)	39 (22.9)

Survey included 170 participants. Numbers may vary by question due to missing data.

#### Comfort teaching harm reduction substance use education

Educator comfort with teaching harm reduction curriculum varied depending on the substance in question, with 86.5% identifying that they would be comfortable teaching about alcohol, 83.5% for cannabis, and 55.9% for unregulated substances. The majority (77.2%) reported that they would be comfortable supporting a student who had consumed cannabis on school grounds; however, that number dropped to 55% when the substance was unregulated. Encouragingly, only 7.7% of educators believed that reducing youth harms from substance use was exclusively the responsibility of the family. While 46.7% worried that providing substance use education and supports to students might contribute to personal stress or burnout for the educator, 78.1% indicated that such involvement would provide them job satisfaction (Table 3).

**Table 3:** Comfort teaching harm reduction substance use education.

	Response N (%)			
Survey item	Strongly agree	Agree	Disagree	Strongly disagree
I would be comfortable providing support to a student who had consumed cannabis on school grounds.	62 (37.1)	67 (40.1)	25 (15.0)	13 (7.8)
I would be comfortable providing support to a student who had consumed an unregulated substance (eg., cocaine, LSD) on school grounds:	43 (25.7)	49 (29.3)	44 (26.3)	31 (18.6)
Preventing harm associated with substance use in youth is exclusively the responsibility of the family.	1 (0.6)	12 (7.1)	101 (59.4)	56 (32.9)
I fear that becoming involved in providing substance use supports to students could lead to me experiencing stress or burnout.	19 (11.2)	60 (35.5)	75 (44.4)	15 (8.9)
Educating students in my school about harm reduction would provide job satisfaction to me as an educator.	40 (24.2)	89 (53.9)	32 (19.4)	4 (2.4)

Survey included 170 participants. Numbers may vary by question due to missing data.

### Needs in order to teach harm reduction cannabis education

#### Perceived need for harm reduction teacher training

A high proportion of educators (67.6%) were confident that they could recognize if a student was under the influence of cannabis. Educators were less sure about how to respond to such an event, with only 39.3% indicating that for cannabis use on school grounds, the appropriate process to follow was clear. Only 7.7% of educators felt their teacher training would allow them to intervene and prevent cannabis-related harms among students. Almost all educators (98.2%) indicated that teachers require training for reducing cannabis-related harms, and 88.5% expressed a personal interest in such training (Table 4).

**Table 4:** Perceived need for harm reduction teacher training.

	Response N (%)			
Survey item	Strongly agree	Agree	Disagree	Strongly disagree
I have confidence in my ability to identify a student who is under the influence of cannabis.	36 (21.2)	79 (46.5)	49 (28.8)	6 (3.5)
In the event that students are found using cannabis on the school grounds, the appropriate process to follow is clear.	16 (9.5)	50 (29.8)	72 (42.9)	30 (17.9)
My teacher training allows me to intervene to prevent cannabis-related harms among students.	2 (1.2)	11 (6.5)	93 (54.7)	64 (37.6)
Teachers need training for preventing cannabis-related harms.	86 (51.2)	79 (47.0)	2 (1.2)	1 (0.6)
I have an interest in training related to providing cannabis harm reduction education and supports to students.	65 (39.4)	81 (49.1)	16 (9.7)	3 (1.8)

Survey included 170 participants. Numbers may vary by question due to missing data.

### Perceived need for harm reduction student education

Only 13% of educators believed that most students they encountered were knowledgeable about the potential harms of cannabis. Student education on the risks of cannabis use was identified as necessary by 97.6%. Educators agreed that students required further cannabis education regarding mental health effects (97.6%), physical health effects (91.7%), risk of impaired driving (91.1%) and risk of dependence or addiction (87.6%) (Table 5).

**Table 5:** Perceived need for harm reduction student education.

	Response N (%)							
Survey item	Strongly agree	Agree		Unsure		Disagree		Strongly disagree
Most students that I have contact with are aware of the harms associated with cannabis.	0	22 (13.0)		64 (37.9)		63 (37.3)		20 (11.8)
Students need to be further educated on the risks associated with cannabis use.	92 (54.4)	73 (43.2)		2 (1.2)		2 (1.2)		0
Students require further cannabis education in the following areas:*	Mental health effects		Physical health effects		Risk of impaired driving		Risk of dependence/ addiction	
	165 (97.6)		155 (91.7)					
					154 (91.1)		148 (87.6)	

*Note*. Survey included 170 participants. Numbers may vary by question due to missing data.

*Respondents were asked to select all that apply.

### Educator cannabis knowledge

Educators completed several knowledge-testing questions about cannabis and related harms. Performance on these items was relatively low; only 29.4% correctly identified that smoking cannabis could impair driving abilities for six to eight hours. When asked which type of cannabis product would *least* impair driving ability, 49.1% correctly responded: “CBD only.” However, only 4.7% of educators correctly identified that the typical THC content of cannabis extract is 70% THC or higher (Table 6).

**Table 6:** Educator cannabis knowledge.

		Responses N (%)		
Survey question	Correct answer	Correct	Incorrect	I Don’t Know
Smoking or vaping cannabis can impair an individual’s ability to drive for:	6–8 hours	50 (29.4)	60 (35.3)	60 (35.3)
In order to minimize adverse effects, people should choose cannabis products that are:*	Low in THC and high in CBD/ Low in both CBD and THC	96 (56.5)	2 (1.2)	72 (42.4)
Which route of cannabis use would produce the most long-lasting psychoactive effects?	Edibles	76 (45.0)	23 (13.6)	70 (41.4)
What is the typical THC content of cannabis extracts and concentrates?	More than 70% THC	8 (4.7)	52 (30.8)	109 (64.5)
Which type of cannabis product would least impair driving ability?	CBD only	83 (49.1)	23 (13.6)	63 (37.3)

Each question included five possible responses including “I don’t know”. Survey included 170 participants. Numbers may vary by question due to missing data.

*Two responses were accepted as correct

### Preferences for educator training and curriculum materials

When asked how they would like to receive educator harm reduction training, 70.1% of educators were interested in interactive training, 50.9% in instructor-led classes, 34.1% in online courses and 25.7% in training videos. The majority (57.1%) of educators preferred receiving harm reduction curriculum resources online. Educators expressed interest in a range of supplementary curriculum resources, particularly sample class activities (82.0%), e-learning resources (80.2%), educational videos (77.2%) and external speakers (74.9%) (Table 7).

**Table 7:** Preferences for educator training and curriculum materials.

Survey question	N (%)
**How would you like to receive training on harm reduction?** *	
Interactive training (e.g., workshops)	117 (70.1)
Instructor led training (e.g., lectures, seminars)	85 (50.9)
Online courses	57 (34.1)
Training videos	43 (25.7)
Readings or websites	25 (15.0)
Other	3 (1.8)
**How would you like to receive harm reduction curriculum resources?** *	
Online (e.g., email, website)	96 (57.1)
No preference	40 (23.8)
Print copy	26 (15.5)
Other	6 (3.6)
**What type of supplementary curriculum resources would you like to receive for student use?** *	
Sample class activities	137 (82.0)
E-learning resources (e.g., websites, apps, toolkits)	134 (80.2)
Educational videos	129 (77.2)
External speakers (e.g., D.A.R.E, M.A.D.D.)	125 (74.9)
Social media posts	51 (30.5)
Other	6 (3.6)

Survey included 170 participants. Numbers may vary by question due to missing data.

*Respondents were asked to select all that apply.

### Effects of demographic variables on educator perspectives

Educator responses significantly differed by gender, with women expressing greater support for harm reduction approaches and men reporting greater comfort providing support to a student under the influence of cannabis or an unregulated substance (S1 Table). Significant differences were also reported by age group; educators aged 39 and under indicated stronger support for harm reduction and greater interest in receiving training compared to educators aged 40 and over (S2 Table).

Responses significantly differed by years of teaching experience on two items, with educators with 20 or more years of experience expressing more support for the “just say no” approach to substance use education and less interest in harm reduction training, compared to educators with fewer years of experience. Responses significantly differed by community size for just one item; educators working in communities with a population of 5000 or less expressed greater agreement with the statement “preventing harm associated with substance use in youth is exclusively the responsibility of the family,” compared to educators working in communities with a population of 15,000 or more (S3 and S4 Tables).

### Educator descriptors of M.A.D.D. and D.A.R.E.

**Fig 1:**
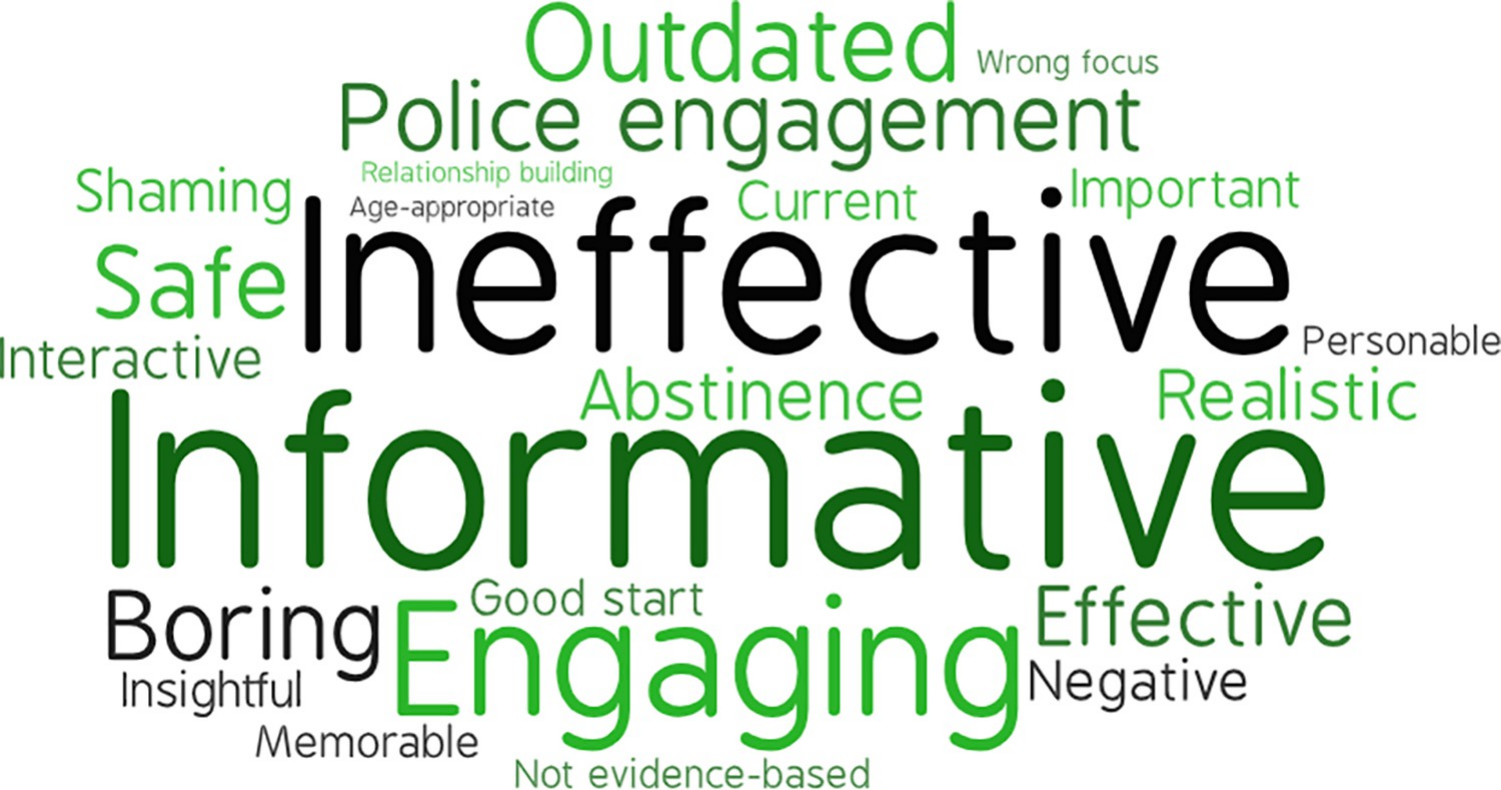
Educator descriptors of D.A.R.E. font size is relative to frequency of reporting.

**Fig 2:**
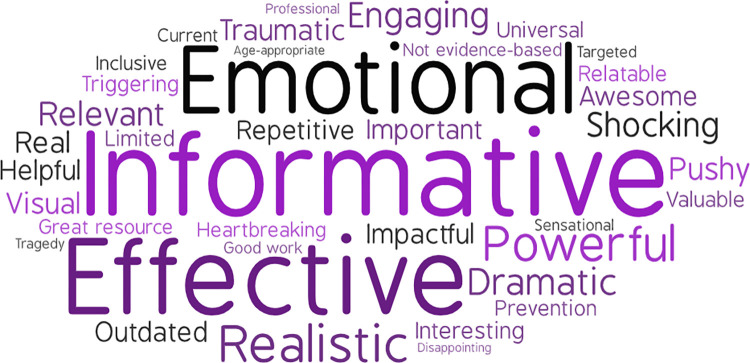
Educator descriptors of M.A.D.D. font size is relative to frequency of reporting.

Reactions to M.A.D.D. and D.A.R.E. were mixed and included a variety of positive and negative descriptors. For the D.A.R.E. program, the words most frequently used to describe the program were “informative,” “ineffective,” “engaging,” and “outdated.” (Fig 1). M.A.D.D. was most often described using the words “realistic,” “effective,” “informative,” and “emotional.” (Fig 2).

### Themes from open-ended survey responses

Open-ended educator responses were qualitatively analyzed inductively and revealed five themes: (i) delivery of substance use education, (ii) harm reduction, (iii) school context, (iv) stigma, and (v) equity, diversity and inclusion. Each theme had a number of sub-themes. The themes, sub-themes and sample quotes are available in Table 8.

**Table 8:** Themes from open-ended survey responses.

Theme	Subtheme	Sample quote
i) Delivery of substance use education	Early education	“Substance use is a huge problem in schools as early as elementary.” -P69W
		“Substance abuse was largely a junior and senior high issue; however, in recent years, I see and hear elementary aged students curious, interested and in some cases knowledgeable about it. I see great value in educating the elementary aged children regarding cannabis as a harm reduction strategy.” -P164W
	Content	“The dangers of cannabis on the developing brain is an important area that many students do not understand.” -P119W
		“That, while it is not a ‘serious’ drug, there are those who fall into using it far too much and their lives are negatively affected.” -P120W
		“That because it’s legal does not mean it’s not harmful (like tobacco, or alcohol).”-P133M
	Evidence-based	“Guest speakers or programs directed at students should be developed and delivered by (or in collaboration with) people with experience using drugs and/or people with direct experience working within harm reduction in the community.” -P187GD“We need realistic, evidence-based, effective and well-developed drug education programs.” -P168W
	Educator needs	“[Substance use education] should be a part of the health curriculum, with adequate training and resources for teachers. Since I don’t consume these products, I am not prepared to teach about them.” -P153W
		“More human resources are needed in schools to address this issue (e.g., psychologists, counselors, administration, social workers, outside agencies).” -P170M
		“Support needs to be in place to achieve any of these things… specifically, people with legitimate training on this topic so they could intervene and help the students.” -P73W
ii) Harm reduction	Educational approach	“I fully support teaching harm reduction in addition to abstinence because it is incredibly naive to think that if we keep preaching abstinence then all kids will follow that message.” -P134W
		“I think a balanced approach to substance use in education is necessary. It is inevitable that some students will use substances. Therefore, we should teach children about harm reduction while still teaching them that abstinence from substance use is the best option.” -P149W
		“I think stimulating conversation around it all in general, and ensuring that this conversation is not based in the traditional model of ‘just say no’, but rather a realistic harm reduction-based approach is a step in the right direction.” -P128M
	Safe supply	“The risk of ordering from non-trustworthy sites online that provide potentially misleading labels surrounding THC content, possible additives added to oils or concentrates.” -P128M
		“Dangers of impurities in product, especially those purchased illegally.” -P186W
		“I can’t even get a naloxone kit in the school.” -P148W
	Driving under the influence of cannabis	“Many students are in school and driving high and if something isn’t soon done, I am afraid that we will see a student hurt or dead.” -P177W
		“Many students think they can use cannabis and still come to school or drive, i.e., a little bit don’t hurt.” -P62M
	Knowledge gaps	“How to recognize and help a friend or peer who is under the influence. How bad a combination of alcohol and marijuana is and how to safely deal with the effects of that combination.” -P65W
		“Not making rookie mistakes of eating too many edibles before feeling the first hit. Everyone seems to make that mistake; a better educated warning could really help students if they do decide to take cannabis.” -P70M
		“The impact it has on the ability of someone to consent to sexual activity—both by user and by the person not using.” -P171W
iii) School context	Normative attitudes	“Students now are so used to the smell of pot in the schools that they do not even comment on it. It is very concerning that it is now a norm rather than an exception and many kids think that as it is a plant then it cannot harm them (this refers to pot, cocaine, mushrooms—all plants so they are fine).” -P171W
		“[Substance use] has become normalized, especially drinking alcohol in our society at large, and students feel a lot of pressure to partake.” -P111W
	Prevalence of substance use	“Vaping is rampant!! They are hiding in the bathrooms and doing it. They even vape in class, it’s the norm. But it is tolerated so they know they can get away with it. As I said earlier, it should be looked at in the same vein as drinking alcohol on school property.” -P195W
		“The vaping issue is starting at younger ages (grade 6) and students do not understand the harms.” -P41W
		“Marijuana use has begun at age 11, and cocaine by junior high, according to my students.” -P111W
iv) Stigma	Medical use	“How it can be used for medicinal purposes under doctor supervision.” -P43W
		“CBD is a valuable medicine to reduce pain. I take it twice daily. Once before school and one in the evening. I tried many regularly available drugs prescribed from my Dr. and nothing has worked to reduce pain like CBD.” -P8W
	Non-judgmental support	“The reality is that some students may not have anyone to talk to and teachers could play an important role in changing their lives/paths. If students feel more support at school and less judgment, they are more likely to continue going.” -P82W
		“We need to understand that students smoke weed, and they need to be able to talk about it. Students are just trying to figure their world out, and it is our responsibility to support them.” -P112W
		“To teach students about where they can go to get help. They should have an easy list of teachers and contacts that they can go to if they are feeling pressured, need help with substances or are worried about substance use with their loved ones.” -P82W
	Stereotypes	“Cannabis can be used responsibly by adults the same as alcohol. Using cannabis does not make you a bad person or a drug addict.” -P136W
		“We need to understand and chat about using it recreationally, just like alcohol. Break the stigma of ‘bad’ use for cannabis.” -P28W
		“Some students may have family members or may themselves use cannabis medically, or another ‘drug’ that often in schools is portrayed as bad, addictive, or harmful. This may cause [the student] some self doubt due to the fact that they are using this drug that they’re hearing horrible things about in school.” -P85W
v) Equity, diversity and inclusion	Community supports	“Most of the kids who I see get in trouble for substance use are kids with rough home lives and [the school] never addresses that depth of it, only punishes the behaviour.” -P140GD
		“It is a very sensitive topic to teach as some students are dealing with the effects of family members abusing substances at home.” -P160W
		“I do NOT think that it should fall to individual teachers to deal with students who are actively on substances during school. These individual interventions should fall to school administration, family, and outside agencies where necessary.” -P149W
	Mental health	“Drug use can be associated with self medicating for stress/anxiety/depression/sexual abuse/ADHD, etc., and therefore it is important for [students] to discuss their difficulties with a trusted adult who may be able to take action on their behalf.” -P166W
		“I feel it would be helpful to acknowledge that many children with ADHD self-medicate to deal with stress but also (likely unknowingly) to feel ‘normal’. If children are aware that this happens perhaps it would encourage them to feel more comfortable asking for help rather than seeking to self- medicate.” -P124W
		“I had a talk yesterday with a student that recently came to our school after 6 months in our community rehab center for youth. He said he started using drugs in grade 7 because he didn’t know how to cope with trauma…More education needs to be done around the ‘whys’ of drug use by youth.” -P119W

P = participant, M = man, W = woman, GD = gender diverse

Educator responses highlighted several considerations regarding the delivery of substance use education. It was suggested that education should start early, as student substance use was noted as a concern as early as elementary school. Educators discussed the content that should be covered in substance use education, such as the cognitive effects of cannabis use and the risk of dependence. It was also emphasized that education should be evidence-based, and content should be informed by people with lived experience with substance use and use a harm reduction approach. A common sentiment was that educators needed additional resources and supports in place in order to confidently teach substance use education.

Harm reduction was a prominent theme, with educators identifying its value within substance use education. Respondents acknowledged that youth substance use is prevalent and suggested that education strategies should take a balanced approach where abstinence is not presented as the only option. Concerns were expressed around safe supply of substances, particularly the risks of youth consuming unregulated cannabis products. Driving under the influence of cannabis was reported as a common concern. Educator responses described youth’s knowledge gaps regarding harm reduction, suggesting that youth need education on topics like recognizing the signs of intoxication and the risks of polysubstance use.

Within the school context, many educators described youth substance use as not only prevalent but also highly normalized. Several respondents described youths’ perceptions that since cannabis was a plant, it was natural and safe to consume. It was also noted that since cannabis and alcohol were legal for adults, youth did not feel that they were harmful. Youth cannabis use and vaping within the school were described as serious problems by many educators, as was social pressure to engage in substance use.

The need to combat the stigma around substance use was another common theme. Educators highlighted the medicinal value of cannabis and the need to teach youth that cannabis use can be beneficial when used responsibly by adults. Further, respondents spoke of the importance of challenging the negative stereotypes around cannabis and the value of providing non-judgmental support to students regarding substance use.

The importance of promoting equity, diversity and inclusion within substance use education emerged as a theme. Educators identified the role that community support plays in youth substance use, acknowledging that not all students have access to the same support within their home environments and larger community. The relationship between mental health and youth substance use was mentioned as an area for education as it was noted that some students may use substances to cope with stress and mental health concerns.

## Discussion

Educators indicated considerable support for harm reduction substance use education and identified the importance of providing students with evidence-based information and supports within the school system. However, many educators also expressed attitudes that aligned with an abstinence approach. These conflicting perspectives about harm reduction and abstinence were not surprising, given that abstinence has been heavily promoted by substance use education programs typically employed in schools, such as the D.A.R.E. program [19, 34]. The concept of abstinence as the desired outcome of substance use education persists despite reports that students consider this approach ineffective [12, 13, 17]. These findings highlight the need to further inform educators of the benefits of offering youth evidence-based, harm reduction substance use education.

It was evident from our research that there is a critical need for educator training around substance use and harm reduction. Educators were more confident in their ability to identify student substance use than in their capacity to intervene effectively. Many educators expressed feeling unprepared to address substance use with students, a sentiment documented in prior qualitative research when Canadian high school teachers reported feeling unequipped and “powerless” to address student cannabis use [28]. In our current study, performance on cannabis knowledge-testing questions suggested that educators were generally uninformed on cannabis. Educators also believed that most students were unaware of the harms of cannabis use. This finding is supported by prior research, as McKiernan and Fleming [12] reported that Canadian youth were uninformed of the cognitive and mental health effects of cannabis use, as well as the risk of driving while under the influence of cannabis. Among our educators, the perceived need for harm reduction substance use education, for educators and students alike, was extremely high.

Educator preferences for harm reduction training and resources were generally in favour of interactive learning and accessible resources. Most educators favoured online access to teaching resources, which was not surprising given the convenience of this method. Less anticipated was that almost three-quarters of educators expressed interest in having external speakers attend the classrooms, such as those sponsored through the D.A.R.E. or M.A.D.D. programs. Opinions on these external programs were quite mixed in our study, with some educators considering them an essential and informative resource and others describing them as ineffective and outdated. This interest in external presentations may have indicated educators’ desire for additional supports in administering substance use education, a need that was brought up frequently in open-ended responses. Educators expressed the need for support from individuals with in-depth knowledge of substance use, such as psychologists and social workers, and presentations from trained professionals. While educators were interested in teaching students to reduce substance-related harms, many expressed that they could not do so on their own.

Our study identified several differences based on participant characteristics, including gender, age, and geographical location. Women indicated greater support for harm reduction, while men expressed greater comfort supporting a student who had consumed cannabis or an unregulated substance. These findings were similar to those reported by Heath et al. [35], who assessed educator knowledge and attitudes toward student self-injury, another prominent health concern that often emerges during adolescence. Heath et al. found that female educators expressed more positive attitudes towards students who engaged in self-injury, while male educators reported greater confidence in addressing an instance of student self-injury. While these parallels are interesting, no other identified studies have examined gender differences in educator approaches to student health concerns. Our study also found that educators under 40 were more supportive of harm reduction education and were more interested in receiving training than those aged 40 and over. This finding may suggest generational differences in attitudes towards substance use due to the long-standing prevalence of the “just say no” approach [34]. Another interesting finding was that those who taught in rural communities were more likely to indicate that preventing the harms associated with substance use was exclusively the family’s responsibility. There is a gap in the literature comparing urban and rural educator attitudes toward teaching substance use education. Future research should further investigate how demographic factors influence educator perspectives toward substance use education so that curriculum can be adapted based on the characteristics of educators in a given population.

Barriers to teaching substance use education were highlighted in our open-ended responses, where educators emphasized limited class time, a lack of resources and supports, and a seemingly insurmountable workload. Johnson et al. [28] reported similar findings, with educators indicating that time constraints and limited resources acted as obstacles to teaching cannabis education to students. Educators in our study often mentioned students’ normative attitudes about substance use, including that cannabis is natural and therefore safe, a perspective also identified by Porath-Waller et al. [13]. Respondents also expressed concern that students were experimenting with substances as early as elementary school. In particular, vaping was described as “rampant” among students, which is alarming given that vaping products often contain contaminants and toxins and may be associated with acute lung injuries among youths [36]. In light of these reports, educators’ suggestions that substance use education should start in elementary school were well justified.

Stigma accompanying cannabis use was identified as an obstacle that must be addressed. Educators expressed that cannabis is not inherently bad, and students should not be made to feel that using cannabis makes them a bad person. Some educators mentioned that cannabis has medical benefits that should not be discounted due to stigmatizing attitudes toward the substance. On another note, it was encouraging that educators in our study mentioned the importance of “meeting students where they are,” in other words, having honest, nonjudgmental conversations with students about substance use. Our educators acknowledged that many youths do experiment with substances and that simply encouraging abstinence is a missed opportunity to offer information and support that could help students prevent possible harm. Accepting the reality of youth substance use and working with them to make positive changes is central to the harm reduction approach [19, 37].

The need to consider equity, diversity and inclusion throughout substance use education was emphasized. Educators recognized that not all students have the same level of support available to them within their community; students facing challenging situations at home are particularly susceptible to substance use and are especially in need of educator support. Students who are impacted by adverse childhood experiences (ACEs) such as abuse, neglect, and unhealthy family dynamics may experience higher rates of smoking, alcohol dependence, depression and physical health issues in adulthood [38]. ACEs that occur at the community level can also impact children as they mature; these community factors may include witnessing violence, experiencing racism, or feeling unsafe in a neighbourhood [39]. As such, it is imperative that educators consider individual differences and adopt a trauma-informed approach, so students can learn in an environment that is safe and supportive [40]. Educators in our study also acknowledged that students may use substances to cope with mental health concerns such as depression, anxiety and attention deficit hyperactivity disorder (ADHD). These concerns are justified as there are reported increased rates of substance use among those with ADHD [41, 42], mood disorders [43, 44] or a history of trauma [45, 46]. Educators in our study also expressed the importance of identifying and addressing possible underlying mental health issues when responding to student substance use.

Our study has several strengths and limitations. One strength was that the sample population was diverse in age, years of teaching experience, and community size. While over three-quarters of participants were women, this proportion was in line with the gender breakdown of NL educators [47]. Another strength was the addition of open-ended questions, which offered a richer understanding of educator perspectives. However, this method of collecting qualitative data had its limitations, as there was no opportunity to probe for more information or clarification around responses. For example, several educators wrote about the prevalence of vaping, but it is unclear whether these comments referred to nicotine or cannabis products. Another limitation to consider is that the sample population consisted almost exclusively of educators working for the NL English school board, which aligns with the school system as 94% of schools in the province are from this school board. However, it is unknown how educator perspectives in the province’s French and private schools may have differed. Despite this, the current sample was representative of the general population of NL educators in terms of age, gender and teaching position [47].

Another consideration is that some survey questions may have been written in favour of harm reduction; as such, there may have been an implicit bias toward harm reduction within the survey items. Further, the survey was developed specifically for this study and was not validated in prior research. However, the survey was reviewed and approved by experienced educators, whose input contributed to the content validity of the measure. We did not correct for multiple comparisons during statistical analysis, as such corrections are not required in exploratory research [48]. The current findings are to be interpreted within a descriptive context. Finally, the voluntary nature of the survey should be noted and the subsequent potential for self-selection bias. Given that many participants may have completed the study out of personal interest in the topic, their attitudes may have differed from those of the general educator population. Future research, including educators from French and private schools, as well as a more diverse sample in terms of gender identity and cultural background, would help produce more generalizable findings.

## Conclusion

Our current study provided insight into grades 4 to 12 educator perspectives toward harm reduction cannabis-focused substance use education. Our results indicated that while educators were receptive to harm reduction approaches, many felt unequipped to translate them into practice in the classroom. Educators expressed an urgent need for training on harm reduction substance use education approaches to effectively teach and support students on this topic. This information on educator perspectives is meaningful as it will help inform the approach to harm reduction substance use education, allowing educators to help students make safe and informed choices.

## Supporting information

S1 Appendix Survey questions. (PDF)

S1 Table Significant independent-samples t-tests for gender. (PDF)

S2 Table Significant independent-samples t-tests for age group. (PDF)

S3 Table Significant one-way ANOVAs for years of teaching experience and community size. (PDF)

S4 Table Games-Howell post-hoc tests for significant ANOVAs. (PDF)
