# Supplementary material for: A survey of educator perspectives toward teaching harm reduction cannabis education
Source: PLoS One. 2024 May 8;19(5):e0299085. doi: 10.1371/journal.pone.0299085 (PMC11078393; doi:10.1371/journal.pone.0299085)
Supplement: S1 Appendix — (PDF) [file pone.0299085.s001.pdf]

## S1 Appendix. Survey questions.

---

Please remember as you complete the survey that you may skip any questions that you would prefer not to answer.

---

This section contains questions about demographic information.

**1. Have you been directly involved in the education of students at the school level in grades 4-12 in Newfoundland and Labrador (NL) at any point since the 2018/19 school year?**

Yes

No

**2. Which of the following school grades have you had direct contact with since 2018/19? (Please select all that apply)**

4-6

7-9

10-12

None of the above

**3. What has been your primary role as an educator since 2018/19?**

Classroom teacher

Principal/vice principal

Guidance counselor

Speciality teacher (e.g., music, physical education)

Teacher-librarian

Teaching and learning assistant

Student assistant

Instructional resource teacher

District support personnel

Other (Please specify) ____

4. **What is your current employment status?**

Permanent

Replacement

Substitute

Retired

On-leave

5. **To what age group do you belong?**

Under 25

25-29

30-39

40-49

50-59

60+

Decline to answer

6. **What is your gender identity?**

Man

Woman

Gender Diverse

Other (Please specify) ____

Decline to answer

7. **How many years of experience do you have as an educator?**

0-5 years

6-10 years

11-15 years

16-20 years

20+ years

8. **What is your teacher certification level?**

Certificate II

Certificate III

Certificate IV

Certificate V

Certificate VI

Certificate VII

Unsure

**9. Which school district do you currently work for?**

Newfoundland and Labrador English School Board

Provincial Francophone School Board of Newfoundland and Labrador

I work for a private school in NL

**10. In what language do you primarily teach?**

French

English

Both French and English

**11. What is the population of the community in which you work?**

Less than 5000 (e.g., Bonavista, Twillingate)

5000 to 15,000 (e.g., Gander, Clarenville)

More than 15,000 (e.g., St. John's, Corner Brook)

**12. How many students are enrolled in the school in which you primarily work?**

Less than 50

51 to 150

151 to 300

301 to 450

451 to 600

More than 600

---

The following sections refer to two different substance use education approaches: abstinence and harm reduction.

Abstinence approach: centers on the idea that avoiding drug use is the only acceptable option for everyone.

Harm reduction approach: recognizes that substance use among some youth is inevitable and focuses on decreasing the harms associated with substance use.

---

Please select the response that best reflects your personal attitude about substance use.

**13. It is possible to use substances without misusing or abusing substances.**

Strongly Agree
Agree
Disagree
Strongly Disagree

**14. Students who use substances should be expected to pursue abstinence.**

Strongly Agree
Agree
Disagree
Strongly Disagree

**15. Students should be given honest information about how to avoid the harms associated with substance use (for example, preventing overdose or health hazards).**

Strongly Agree
Agree
Disagree
Strongly Disagree

**16. Minimizing the risk of harm associated with substances should be discussed with students who seek help for substance use.**

Strongly Agree
Agree
Disagree
Strongly Disagree

**17. Students who use substances should have access to support services within the school system to help prevent harm.**

Strongly Agree

Agree

Disagree

Strongly Disagree

**18. Harm reduction is a practical, realistic approach that does not encourage substance use.**

Strongly Agree

Agree

Disagree

Strongly Disagree

**19. The "just say no" message regarding substance use is effective for many youths.**

Strongly Agree

Agree

Disagree

Strongly Disagree

**20. Access to evidence-based information on substances allows youth to make safer choices.**

Strongly Agree

Agree

Disagree

Strongly Disagree

**21. Abstinence-based education reduces harm associated with substance use among youth.**

Strongly Agree

Agree

Disagree

Strongly Disagree

**22. A harm reduction approach to substance use education can present abstinence to youth as an option without framing it as the only choice.**

Strongly Agree

Agree

Disagree

Strongly Disagree

**23. Teaching youth about safer substance use will encourage them to use substances.**

Strongly Agree

Agree

Disagree

Strongly Disagree

**24. I would be comfortable teaching harm reduction to students regarding the following substances (please select all that apply):**

Alcohol

Cannabis

Unregulated substances (eg., cocaine, LSD)

None of the above

**25. I would be comfortable providing support to a student who had consumed cannabis on school grounds:**

Strongly Agree

Agree

Disagree

Strongly Disagree

**26. I would be comfortable providing support to a student who had consumed an unregulated substance (eg., cocaine, LSD) on school grounds:**

Strongly Agree

Agree

Disagree

Strongly Disagree

**27. Preventing harm associated with substance use in youth is exclusively the responsibility of the family.**

Strongly Agree

Agree

Disagree

Strongly Disagree

**28. I fear that becoming involved in providing substance use supports to students could lead to me experiencing stress or burnout.**

Strongly Agree

Agree

Disagree

Strongly Disagree

**29. Educating students in my school about harm reduction would provide job satisfaction to me as an educator.**

Strongly Agree

Agree

Disagree

Strongly Disagree

---

The following questions focus specifically on cannabis.

**30. I have confidence in my ability to identify a student who is under the influence of cannabis.**

Strongly Agree

Agree

Disagree

Strongly Disagree

**31. In the event that students are found using cannabis on the school grounds, the appropriate process to follow is clear.**

Strongly Agree

Agree

Disagree

Strongly Disagree

**32. My teacher training allows me to intervene to prevent cannabis-related harms among students.**

Strongly Agree

Agree

Disagree

Strongly Disagree

**33. What strategies have you used in managing cannabis-related concerns?** (Open-ended)

**34. Teachers need training for preventing cannabis-related harms.**

Strongly Agree

Agree

Disagree

Strongly Disagree

**35. I have an interest in training related to providing cannabis harm reduction education and supports to students.**

Strongly Agree

Agree

Disagree

Strongly Disagree

---

Based on what you know about cannabis, please answer the following questions.

**36. Smoking or vaping cannabis can impair an individual's ability to drive for:**

30 min-1 hour

1-2 hours

3-5 hours

6-8 hours

I don't know

**37. In order to minimize adverse effects, people should choose cannabis products that are:**

Low in CBD and high in THC

Low in THC and high in CBD

Low in both THC and CBD

High in both THC and CBD

I don't know

**38. Which route of cannabis use would produce the most long-lasting psychoactive effects?**

All routes produce the same effect

Edibles

Smoking

Vaping

I don't know

**39. What is the typical THC content of cannabis extracts and concentrates?**

Less than 30% THC

30-50% THC

51-70% THC

More than 70% THC

I don't know

**40. Which type of cannabis product would <u>least</u> impair driving ability?**

All product types are equally impairing

CBD only

THC only

Both THC and CBD

I don't know

---

Please select the response that best reflects your personal attitude about cannabis education.

**41. Most students that I have contact with are aware of the harms associated with cannabis.**

Strongly Agree

Agree

Unsure

Disagree

Strongly Disagree

**42. Students need to be further educated on the risks associated with cannabis use.**

Strongly Agree

Agree

Unsure

Disagree

Strongly Disagree

**43. Students require further cannabis education in the following areas (please select all that apply):**

Mental health effects

Physical health effects

Risks of impaired driving

Risk of dependence/addiction

None of the above

**44. What other aspect(s) of cannabis use should be addressed in the curriculum?** (Open ended)

---

In this section we will be asking about your preferences regarding educator training, receiving curriculum, and supplementary resources.

**45. How would you like to receive training on harm reduction? (Please select all that apply)**

Instructor led training (e.g., lectures, seminars)

Interactive training (e.g., workshops)

Online courses

Training videos

Readings or websites

Other (Please specify) ____

**46. How would you like to receive harm reduction curriculum resources?**

Print copy

Online (e.g., email, website)

No preference

Other (Pease Specify) ____

**47. What type of supplementary curriculum resources would you like to receive for student use? (Please select all that apply)**

Educational videos

E-learning resources (e.g., websites, apps, toolkits)

External speakers (e.g., DARE, MADD.)

Social media posts

Sample class activities

Other (Please specify) ____

---

The following section contains questions about Mothers Against Drunk Driving (M.A.D.D.) and Drug Abuse Resistance Education (D.A.R.E.).

**48. Since 2018/19, has the school that you work at hosted educational presentations by D.A.R.E?**

Yes

No

Unsure

**49. Provide three words or phrases that describe your perceptions of the D.A.R.E. program.** (Open-ended)

**50. Since 2018/19, has the school that you work at hosted educational presentations by M.A.D.D?**

Yes

No

Unsure

**51. Provide three words or phrases that describe your perceptions of the M.A.D.D. program.** (Open-ended)

---

**52. Please add any final thoughts that you would like to share regarding cannabis education in the school system.** (Open-ended)

---

**53. Would you like to enter the prize draw for a chance to win one of four $50 Amazon gift cards? If you click "yes," you will be redirected to a separate link where your contact information will be collected. Your survey responses will still remain anonymous.**

Yes

No

---

Thank you for participating in this survey! Your contribution is greatly appreciated.
