# Supplementary material for: A survey of educator perspectives toward teaching harm reduction cannabis education
Source: PLoS One. 2024 May 8;19(5):e0299085. doi: 10.1371/journal.pone.0299085 (PMC11078393; doi:10.1371/journal.pone.0299085)
Supplement: S1 Table — (PDF) [file pone.0299085.s002.pdf]

S1 Table. Significant independent-samples t-tests for gender.

Survey Item	Males (*n* = 35)		Females (*n* = 130)		*t*	*p*	*d*
	M	***SD***	***M***	***SD***			
Harm reduction is a practical, realistic approach that does not encourage substance use.	1.83	0.79	1.57	0.63	2.03	.044*	0.39
Teaching youth about safer substance use will encourage them to use substances.	2.80	0.76	3.10	0.66	-2.32	.022*	-0.44
I would be comfortable providing support to a student who had consumed cannabis on school grounds:	1.65	0.88	2.04	0.91	-2.25	.026*	-0.43
I would be comfortable providing support to a student who had consumed an unregulated substance (e.g., cocaine, LSD) on school grounds:	2.06	1.07	2.51	1.03	-2.23	.027*	-0.43
In the event that students are found using cannabis on the school grounds, the appropriate process to follow is clear.	2.43	0.81	2.77	0.87	-2.05	.042*	-0.39

Lower score indicates greater agreement with survey item.

*p < .05, ***p* < .01, ****p* < .001
