# Supplementary material for: A survey of educator perspectives toward teaching harm reduction cannabis education
Source: PLoS One. 2024 May 8;19(5):e0299085. doi: 10.1371/journal.pone.0299085 (PMC11078393; doi:10.1371/journal.pone.0299085)
Supplement: S2 Table — (PDF) [file pone.0299085.s003.pdf]

S2 Table. Significant independent-samples t-tests for age group.

**Survey Item**	**39 and under (*n* = 89)**		**40 and over (*n* = 81)**		***t***	***p***	***d***
	M	***SD***	***M***	***SD***			
Minimizing the risk of harm associated with substances should be discussed with students who seek help for substance use.	1.19	0.40	1.41	0.61	-2.78	.006**	-0.43
Harm reduction is a practical, realistic approach that does not encourage substance use.	1.45	0.54	1.79	0.74	-3.39	<.001***	-0.53
The “just say no” message regarding substance use is effective for many youths.	3.08	0.73	2.83	0.84	2.11	.037*	0.32
Abstinence-based education reduces harm associated with substance use among youth.	2.89	0.73	2.57	0.83	2.58	.011*	0.40
A harm reduction approach to substance use education can present abstinence to youth as an option without framing it as the only choice.	1.39	0.51	1.69	0.65	-3.29	.001**	-0.51
In the event that students are found using cannabis on the school grounds, the appropriate process to follow is clear.	2.85	0.84	2.51	0.89	2.55	.012*	0.39
My teacher training allows me to intervene to prevent cannabis-related harms among students.	3.38	0.53	3.19	0.73	2.03	.044*	0.31
I have an interest in training related to providing cannabis harm reduction education and supports to students.	1.56	0.58	1.94	0.78	-3.50	<.001***	-0.55

Lower score indicates greater agreement with survey item.

*p < .05, ***p* < .01, ****p* < .001
