# Supplementary material for: A survey of educator perspectives toward teaching harm reduction cannabis education
Source: PLoS One. 2024 May 8;19(5):e0299085. doi: 10.1371/journal.pone.0299085 (PMC11078393; doi:10.1371/journal.pone.0299085)
Supplement: S3 Table — (PDF) [file pone.0299085.s004.pdf]

S3 Table. Significant one-way ANOVAs for years of teaching experience and community size.

**Survey Item**	***F***	***p***	η^2
Years of Teaching Experience			
The “just say no” message regarding substance use is effective for many youths.	2.83	.026*	.065
I have an interest in training related to providing cannabis harm reduction education and supports to students.	4.25	.003**	.096
Community Size			
Preventing harm associated with substance use in youth is exclusively the responsibility of the family.	5.44	.005**	.062

*p < .05, ***p* < .01, ****p* < .001
