# Supplementary material for: A survey of educator perspectives toward teaching harm reduction cannabis education
Source: PLoS One. 2024 May 8;19(5):e0299085. doi: 10.1371/journal.pone.0299085 (PMC11078393; doi:10.1371/journal.pone.0299085)
Supplement: S4 Table — (PDF) [file pone.0299085.s005.pdf]

S4 Table. Games-Howell post-hoc tests for significant ANOVAs.

**Survey Item**	**Comparison**		**Mean Difference**	***p***	**95% Confidence Interval**	
	**Variable Level**	**Variable Level**			**Lower bound**	**Upper bound**
Years of Teaching Experience						
The "just say no" message regarding substance use is effective for many youths.	20+	0-5	-0.60	.011*	-1.10	-0.10
		6-10	-0.22	.719	-0.72	0.28
		11-15	-0.27	.616	-0.81	0.26
		16-20	-0.42	.271	-1.02	0.17
I have an interest in training related to providing cannabis harm reduction education and supports to students	20+	0-5	.60	.004**	0.15	1.05
		6-10	.37	.210	-0.11	0.84
		11-15	.50	.033*	0.03	0.98
		16-20	.19	.922	-0.47	0.85
Community Size						
Preventing harm associated with substance use is exclusively the responsibility of the family	Less than 5,000	5000-15,000	-0.11	.682	-0.43	0.21
		15,000+	-0.34	.004**	-.058	-0.09

*p < .05, ***p* < .01, ****p* < .001
